# Application of Next-Generation Sequencing for the Determination of the Bacterial Community in the Gut Contents of Brackish Copepod Species (*Acartia hudsonica*, *Sinocalanus tenellus*, and *Pseudodiaptomus inopinus*)

**DOI:** 10.3390/ani11020542

**Published:** 2021-02-19

**Authors:** Yeon-Ji Chae, Hye-Ji Oh, Kwang-Hyeon Chang, Ihn-Sil Kwak, Hyunbin Jo

**Affiliations:** 1 Department of Environmental Science and Engineering, Kyung Hee University, Yongin 1732, Korea; 016399co@naver.com (Y.-J.C.); chang38@khu.ac.kr (K.-H.C.); 2 Department of Ocean Integrated Science, Chonnam National University, Yeosu 59626, Korea; inkwak@hotmail.com; 3 Institute for Environment and Energy, Pusan National University, Busan 46241, Korea

**Keywords:** metabarcoding, gut bacterial composition, brackish reservoir, operational taxonomic unit (OTU) assignment

## Abstract

**Simple Summary:**

Copepods are important components of marine coastal food chains, supporting fishery resources by providing prey items mainly for fish. Copepods interact with small microorganisms via feeding on phytoplankton. DNA methods can determine the gut contents of copepods and provide important information regarding how copepods interact with phytoplankton and bacteria. In the present study, we designed a method for extracting the gut content DNA from small-sized copepods that are important in coastal and brackish areas. Based on DNA analyses, Rhodobacteraceae, which is common in marine waters and sediments, was most abundant in the gut contents of the three copepod species (*Acartia hudsonica, Sinocalanus tenellus*, and *Pseudodiaptomus inopinus*). However, the detailed composition of bacteria was different among species and locations. The results suggested that environmental variables and species-specific feeding behaviour can affect the gut bacterial community. The bacteria play an important role in digestion and in the overall degradation and release of metabolites to the outside water. Further analyses with advanced methods regarding DNA isolation from small microorganisms and identification skills using a DNA library for better understanding of biological interactions and matter cycling in marine food webs are required.

**Abstract:**

The gut bacterial communities of copepods can affect metabolic processes, and consequently, their activity can be related to the release of organic substances to the environment. Hence, they are important for organic matter cycling in marine coast food webs. However, information regarding the variation in gut bacterial communities based on copepod species and environmental variations is limited. We analysed the differences in gut bacterial communities from dominant copepod species, i.e., *Acartia hudsonica, Sinocalanus tenellus*, and *Pseudodiaptomus inopinus*, in a brackish reservoir. The core bacteria among the copepod species and locations consisted of the following main operational taxonomic units (OTUs): *Novosphingobium capsulatum* and the family Rhodobacteraceae belonging to Alphaproteobacteria, which is abundant in seawater and freshwater aquatic ecosystems as a zooplankton-associated bacterial community. The bacterial community composition of each copepod (except the core species) showed high variability. The bacterial community diversity differed depending on the copepod species and the sites’ environmental conditions, especially salinity, e.g., compositional variations in the bacterial community of *P. inopinus* were high at sites with low salinity. Therefore, the gut bacterial community of each copepod species responds differently to the environment.

## 1. Introduction

Copepods are a dominant zooplankton community in coastal and marine waters and play an important role in microbial and grazing food webs. From a bottom-up perspective, copepods are closely related to ecosystem services as a critical food resource for fish [1]. Copepods release dissolved and particulate organic carbon when they feed and defecate, which contribute nutrients to support microorganisms [2,3]. Copepod-associated bacterial communities simultaneously influence copepod nutrient uptake efficiency by increasing the absorptive area and degrading toxic substances released by cyanobacteria [4,5,6]. Bacterial communities in the acidic and anaerobic environments in the copepod digestive system (gut bacterial communities) are involved in the metabolic processing of nutrients ingested by copepods, including denitrification, methanogenesis, iron circulation, and phosphorus metabolism [7,8]. Interaction between copepods and gut bacteria affects the organic matter cycling in aquatic ecosystems [9,10]. However, a comprehensive understanding of the biological interaction between copepods and gut bacteria is elusive because of the low-resolution information regarding bacteria related to the numerous copepod species, despite the important ecological functions of these bacterial communities.

The introduction of DNA technology to microbiome analyses in both aquatic and terrestrial ecosystems has been a driving force for the study of small-sized organisms and their associated bacteria [11,12,13,14,15]. The copepod community and its associated bacterial community was also more actively analysed through the application of DNA analysis techniques; however, previous studies on copepod-associated bacteria have been primarily conducted at the laboratory scale (e.g., incubation) [16,17,18]. Hence, there are limitations in the understanding of copepod responses to natural habitats affected by various environmental factors simultaneously. For copepod gut bacterial community analyses, a method of dissecting the gut from individual specimens has been commonly used [19]. This method has been used for large species such as *Calanus* (2–3 mm of prosomal length); however, it is difficult to apply it to small-sized species [20,21]. Although the DNA analysis methods of the gut contents of small zooplankton (rotifers, under 400 μm) have been proposed [22], zooplankton species-specific differences, such as exoskeleton hardness, must be considered, and methods that can be applied to small-sized copepod species (700 μm–1 mm of prosomal length) are still required [23].

In the present study, we designed a DNA extraction method for the copepod gut without dissection and analysed the composition of copepod gut bacterial communities in a semi-closed estuarine ecosystem. Compared with open systems (e.g., the coast), physicochemical properties such as seawater input, water current, and overall salinity distribution were regulated by the reservoir because of the construction of a dyke with sluice gates. This regulated environment allowed the tracking of relatively stable environmental gradients [24], and was suitable for monitoring copepod distribution, interaction with different environments, and the responses of the gut bacterial community. We extracted the gut contents from three copepod species (*Acartia hudsonica*, *Sinocalanus tenellus*, and *Pseudodiaptomus inopinus*) that had different adaptations to salinity. Samples were collected from three sites located along a salinity gradient (from near freshwater input to seawater input through the sluice gate), and next-generation sequencing (NGS) was performed to determine the bacterial composition differences.

## 2. Materials and Methods

### 2.1. Sampling Site and Period

**Figure 1:**
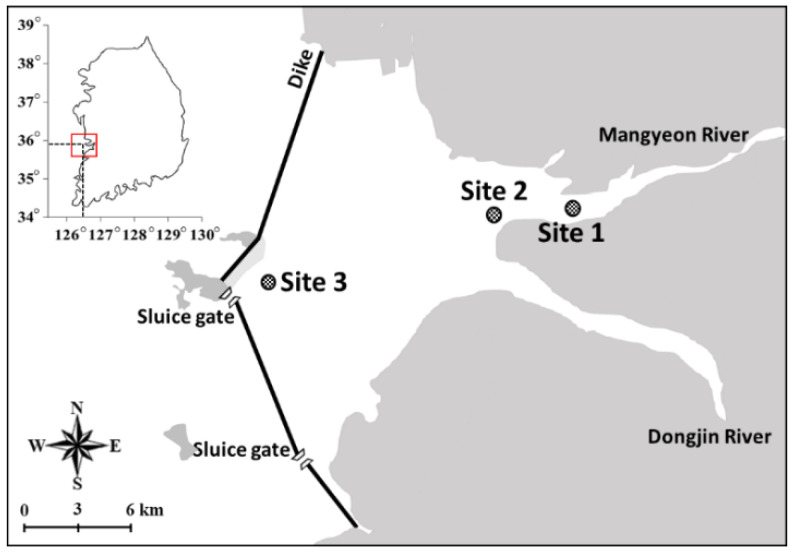
Study sites within the Saemangeum reservoir: the Mangyeong River mouth (Site 1), the point of maximum freshwater inflow to the reservoir (Site 2), and a site close to the northernmost sluice gate (Site 3).

The Saemangeum reservoir (35°50′17.1″ N, 126°35′36.6″ E) was constructed by blocking the Saemangeum coast with dykes. The dyke-side bay is a semi-closed coastal ecological system, with the flux of seawater controlled by two sluice gates on the southern part of the dykes [25]. Sluice gates are opened to allow seawater inflow because excessive nutrients from agricultural and livestock sewage flowing from the Mangyeong and Dongjin rivers adjacent to the Saemangeum reservoir contribute to eutrophication [26]. Simultaneous inflows of freshwater and seawater create a salinity gradient in the reservoir and a brackish ecosystem [27]. We selected three different sites within the reservoir for study: the Mangyeong River mouth (Site 1; 35°51′29.74″ N, 126°41′04.04″ E); the point of maximum freshwater inflow to the reservoir (Site 2; 35°51′04.07″ N, 126°38′00.55″ E), and a site close to the northernmost sluice gate (Site 3; 35°49′08.07″ N, 126°30′11.27″ E) (Figure 1).

On 11 May 2020, we analysed water quality and plankton composition to identify the environmental conditions of each site and collected samples to be used in the gut bacterial community analysis of each site’s dominant copepod species.

### 2.2. Environmental Characteristics: Water Quality and Plankton Compositions

We measured basic water quality parameters and analysed the plankton community to understand the environmental characteristics of each site. The water quality was measured from the surface layer (0–0.5 m) of each study site. Temperature (°C), pH, electrical conductivity (S/m), and salinity (‰) data were collected with a Horiba (u-20) on site. Dissolved oxygen (mg/L), chemical oxygen demand (mg/L), total nitrogen (mg/L), total phosphorus (mg/L), and chlorophyll-*a* (ug/L) were analysed in the laboratory following Water Pollution Process Test Standards [28] and Marine Environmental Process Test Standards [29].

Phytoplankton samples were collected 1 L of raw water from the surface layer (0–0.5 m) into polyethylene bottles and fixed at a final concentration of 4–5% Lugol’s solution at the site. We prepared fixed samples as immersion specimens via sedimentation for more than 24 h and the supernatant solution was removed with a siphon. Algae were identified under 600–1000× magnification with an optical microscope (Zeiss Axio Imager A2, Jena, Germany). Suspended diatoms were washed using the KMnO_4_ method [30] and were then made into permanent samples. These specimens were identified at a magnification of 1000–2500×.

Zooplankton samples were collected to identify and quantify the populations of brackish copepod species at each study site. At shallow water Site 1, we towed a net three times, 3 m each with 60 μm pore size and 0.2 m diameter horizontally for zooplankton sampling (filtered volume: 0.283 m^3^). In the case of the Sites 2 and 3 inside the Saemangeum reservoir, we measured water depths of each site first, and then the net (Kitahara zooplankton net; pore size 100 μm, diameter 0.3 m) were vertically towed from bottom to the surface based on the water depths. We towed the net once at Site 2 and twice at Site 3, considering the filtered volume according to the water depth of each site (filtered volume: 0.636 m^3^ and 1.590 m^3^ respectively). Zooplankton samples were fixed at a final concentration of 4–5% with formalin at the site. We extracted 5 mL of sub-sample from each specimen concentrated and identified under an optical microscope (Olympus CKX 41, Tokyo, Japan) at 100× magnification in the laboratory.

### 2.3. Sample Collection and Treatment for DNA Analysis

We collected copepod samples for analysing copepod-associated bacterial communities, especially gut microorganisms. We repeated the zooplankton collection as described above for each site until enough mass was collected for analysis. The collected samples were transported to the laboratory in refrigerated storage and kept frozen until DNA analysis was performed.

Copepod individuals were selected before DNA extraction for pretreatment to eliminate extracellular DNA attached to the exoskeleton. Individuals of *A. hudsonica*, *S. tenellus*, and *P. inopinus* were sorted under a dissecting microscope and stored separately in glass vials filled with 60% ethanol to prevent cross-contamination among individuals (*n* = 3, respectively). The exoskeleton of each copepod individual was exposed to commercial bleach diluted 2.5% for 2 min and then washed three times with distilled water to avoid any effects from the bleach remaining on the skeleton [22]. Pre-treated copepod individuals were stored individually in 2 mL microtubes.

When collecting copepod samples in the field, we simultaneously collected 500 mL of raw water to verify the effectiveness of the pretreatment process. Raw water samples from each site were filtered through a 20 μm net to remove suspended cells, and commercial bleach was added at a final concentration of 2.5%. After 2 min, commercial samples with bleach were filtered through GF/F paper (Whatman^TM^, Little Chalfont, UK) and washed three times with distilled water. All filter papers were stored in 2 mL microtube individually. During these processes, including the pretreatment process, we used bleach sterilised gloves and instruments sterilised with an autoclave and ethanol to minimise contamination from the surrounding environment.

### 2.4. DNA Extraction and 16S rRNA Amplicon Library Generation

DNA analysis from extraction to the 1st polymerase chain reaction (PCR) was conducted on a clean bench with bleach sterilised gloves and instruments sterilised with an autoclave and ethanol. Genomic DNA was isolated from the copepods, and filter paper samples were obtained using a DNeasy Blood and Tissue Kit (Qiagen, Hilden, Germany) following the manufacturer’s instructions except for the following differences: (1) twice the amount of buffer ATL (360 μL) and proteinase K (40 μL) was used on the filter paper samples to increase DNA extraction, (2) the DNA eluting step was repeated, and (3) the final amount of DNA extracted from each sample was 50 μL. The extracted DNA was stored at −20 °C.

Extracted DNA for sequencing was prepared according to the Illumina 16S Metagenomic Sequencing Library protocols (San Diego, CA, USA). DNA quantity, quality, and integrity were measured by PicoGreen (Thermo Fisher Scientific, Waltham, MA, USA) and VICTOR Nivo Multimode Microplate Reader (PerkinElmer, Waltham, MA, USA). Amplification used an AccuPower Hot Start PCR PreMix (Bioneer, Korea) with genomic DNA and primers in a final volume of 20 μL. We used bacterial primers targeting the V3/V4 region of 16S rRNA including an adapter sequence for Illunima. Forward: 5′-TCGTCGGCAGCGTCAGATGTGTATAAGAGACAGCCTACGGGNGGCWGCAG-3′ and reverse: 5′-GTCTCGTGGGCTCGGAGATGTGTATAAGAGACAGGACTACHVGGGATCTAATCC-3′ [31]. A gradient PCR was performed with a thermal cycler (Bio-Rad, Hercules, CA, USA) under the following conditions: initial denaturation at 95 °C for 3 min, followed by 30 cycles of denaturation at 95 °C for 30 s, annealing at 55–65 °C for 30 s, elongation at 72 °C for 30 s, and a final extension at 72 °C for 5 min. After extension, the reactions were held at 4 °C. Amplification products were separated in 1.5% gel electrophoresis.

After amplification, genomic DNA was pooled by site and sample type (copepods *A. hudsonica*, *S. tenellus*, and *P. inopinus*, and commercial bleach-treated water samples). As a second process, to produce indexing PCR, the first PCR product was subsequently amplified with one cycle of 3 min at 95 °C, 8 cycles of 30 s at 95 °C, 30 s at 55 °C, 30 s at 72 °C, and a final step of 5 min at 72 °C. A subsequent limited-cycle amplification step is performed to add multiplexing indices. The final products are normalized and pooled using the PicoGreen (Thermo Fisher Scientific, Waltham, MA, USA), and the size of libraries are verified using the LabChip GX HT DNA High Sensitivity Kit (PerkinElmer, Waltham, MA, USA) and NGS analysis, including index PCR, was completed by Macrogen Co. (Seoul, Korea). The sequencing library is prepared by random fragmentation of the DNA or cDNA sample, followed by 5’ and 3’ adapter ligation. Alternatively, “tagmentation” combines the fragmentation and ligation reactions into a single step that greatly increases the efficiency of the library preparation process. Adapter-ligated fragments are then PCR amplified and gel purified. The PCR products were sequenced using the MiSeq™ platform (Illumina, San Diego, CA, USA) from commercial service (Macrogen Inc., Seoul, Korea). Obtained sequences were deposited in DRYAD.

### 2.5. Data Analyses

Raw reads were trimmed with CD-HIT-OTU and chimeras were identified and removed using rDNATools. For paired-end merging, FLASH (Fast Length Adjustment of Short reads) version 1.2.11 was used. Merged reads were processed and were clustered into OTUs using a bioinformatic algorithm, UCLUST [32], at a 97% OTU cut-off value (352 OTUs in gamma-diversity). Taxonomy was assigned to the obtained representative sequences with BLAST (Reference DB: NCBI—18S) [33] using UCLUST [32]. For the aforementioned processes of BLAST and UCLUST, we used an open-source bioinformatics pipeline for performing microbiome analysis, QIIME version 2 [34]. We classified each OTU according to identity percentage (%): species level with ≥97%, genus level with ≥90%, and family level with ≥84% reads with less than 84% identity were excluded. OTUs detected from commercial bleach-treated water samples were not included with copepod samples because these OTUs made it difficult to identify gut bacterial communities from pre-treated copepod samples (Table S1).

OTU data were statistically analysed based on the microbiome package in R Studio (3.6.3 version) [35]. We selected the following alpha diversity indices: observed species: count of OTUs in each sample; Chao-1 index: estimated diversity from abundance data; Shannon’s diversity index, and Simpson’s dominance and evenness indices; low-abundance index: the concentration of low-abundance taxa below the indicated detection thresholds. These indices were used to compare the change tendencies of each copepod gut bacterial community diversity by site. Principal coordinate analysis (PCoA) based on the Bray-Curtis dissimilarity was conducted to show difference in microbial composition between samples among copepod gut bacterial communities by species and study sites. Core bacterial taxa shared between the sites were analysed to identify bacteria that played the role of key species within the gut bacterial community of copepods collected at each site (with 0.001 detection probability in at least 90% of samples and 0.75 prevalence).

## 3. Results and Discussion

### 3.1. Environmental Conditions and the Copepod Community Composition by Microscopic Examination

The study sites in the Saemangeum reservoir showed differences in water quality parameters, with increasing salinity and electrical conductivity from Sites 1 to 3. The water temperature at Site 1 was high compared with that at Sites 2 and 3. No noticeable differences in pH were observed among the sites. Dissolved oxygen and chlorophyll-*a* (Chl-*a*) concentration were the highest at Site 2, and decreased in the order of Sites 2, 1, and 3. Chemical oxygen demand (COD) showed concentrations from high to low for Sites 2, 3, and 1, while total phosphorus showed the opposite trends to COD. Total nitrogen decreased from Sites 1 to 3, with Site 3 exhibiting a low concentration (Table 1). These spatial gradients of water quality parameters seem to have been formed by the inflow of freshwater with high nutrients from the Mangyeong River into the reservoir due to the semi-closed coastal characteristics. Bacillariophyta was dominant, particularly at Site 3, where salinity was relatively high, showing a high proportion (~93%) (Table 1).

**Figure 2:**
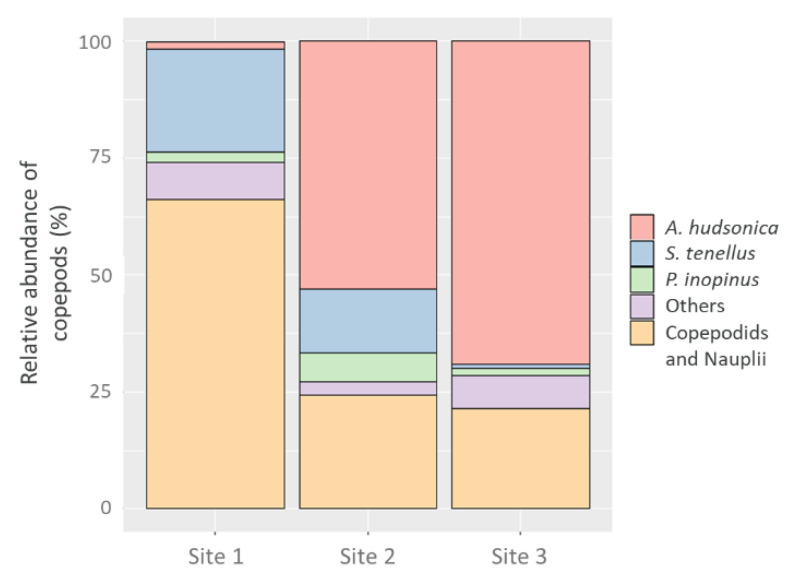
Relative abundance of copepod communities (%) by microscopic examination.

Copepod community composition was different at Site 1, where the proportion of copepods and nauplii (66.14%; 24.57 ind/L) was relatively higher than that of copepod adults. *S. tenellus* showed the highest proportion among adults (22.05%; 8.19 ind/L) and other copepod species contributed 7.87% (2.92 ind/L). *A. hudsonica* and *P. inopinus* accounted for small proportions of less than 3% (1.57%; 0.58 ind/L and 2.36%; 0.88 ind/L, respectively). The proportions of copepodids and nauplii were lower in Sites 2 and 3 compared with Site 1 (24.24%; 2.52 ind/L and 21.47%; 0.88 ind/L, respectively). *A. hudsonica* was the dominant species among the copepod adults (53.03%; 5.50 ind/L). *S. tenellus* (13.64%; 1.42 ind/L) and *P. inopinus* (6.06%; 0.63 ind/L) also made notable contributions at Site 2. Other copepod species exhibited low abundance of 3.03% (0.31 ind/L). The copepod community at Site 3 consisted primarily of *A. hudsonica,* accounting for 69.02% (2.83 ind/L) of the copepod community followed by copepodids and nauplii. Other copepod species, including *Calanidae* spp., *Corycaeus* spp., *Centropages abdominals*, *C. tenuiremis*, *Oithona* spp., and *Paracalanus parvus s.l*. contributed 7.06% (0.29 ind/L). *S. tenellus* and *P. inopinus* were less abundant at 2% (0.92% and 1.53%, respectively) (Figure 2).

*A. hudsonia* is a common estuarine calanoid copepod and its optimal salinity is from 11 to 36‰ [36,37,38]. *S. tenellus* and *P. inopinus* are representative brackish calanoid copepod species and their optimal salinities are approximately 10‰ [39,40]. Therefore, the distribution patterns of targeted copepod species in Saemangeum reservoir were closely related to the preferred habitat environment, especially salinity.

### 3.2. Comparison of Copepod Gut Bacterial Communities Based on the NGS Analysis

Amplicon sequencing produced an average of 57,352 ± 12,348 reads per sample. A total of 106 high-abundance operational taxonomic units (OTUs) were identified after the removal of OTUs with <100 reads. The final number of reads was 56,943 ± 12,560.

#### 3.2.1. Community Composition and Diversity Indices

**Figure 3:**
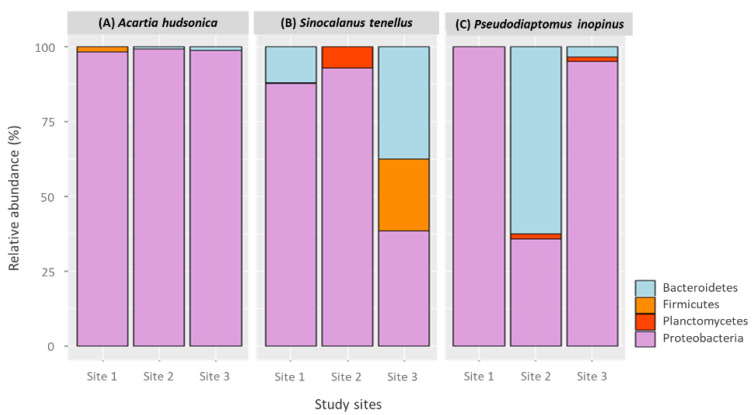
Relative operational taxonomic unit (OTU) abundance of copepod gut bacterial communities (%) based on phyla—(**A**) *Acartia hudsonica*, (**B**) *Sinocalanus tenellus*, and (**C**) *Pseudodiaptomus inopinus.*

The gut bacterial community of *A. hudsonica* displayed the highest proportion of phylum Proteobacteria (more than 98%), regardless of habitat. The phyla Firmicutes at Site 1 (1.6%) and Bacteroides at Sites 2 and 3 (0.9% and 1.3%, respectively) were also identified; however, they accounted for very little of each bacterial community (Figure 3A). 

For *S. tenellus*, species in the phylum Proteobacteria were highly abundant at Sites 1 and 2 (87.6% and 92.9%, respectively). Species in the phyla Bacteroidetes (11.9%) and Firmicutes (0.5%) accounted for the remaining community; the phylum Planctomycetes (7.1%) accounted for the remaining bacteria at Site 2. The gut bacterial community of *S. tenellus* at Site 3 consisted of species in the phyla Proteobacteria (38.3%) and Bacteroidetes (37.5%), with a relatively lower contribution from Firmicutes (24.2%) (Figure 3B).

No bacterial species outside the phylum Proteobacteria were identified in the gut bacterial community of *P. inopinus* at Site 1. Conversely, their gut bacterial community at Site 2 showed a predominance of Bacteroidetes (62.7%), followed by Proteobacteria (35.8%) and Planctomycetes (1.5%). The phylum Proteobacteria was again dominant at Site 3, accounting for 95.1% of bacterial species; the phyla Bacteroidetes (3.5%) and Planctomycetes (1.4%) made small contributions (Figure 3C).

**Figure 4:**
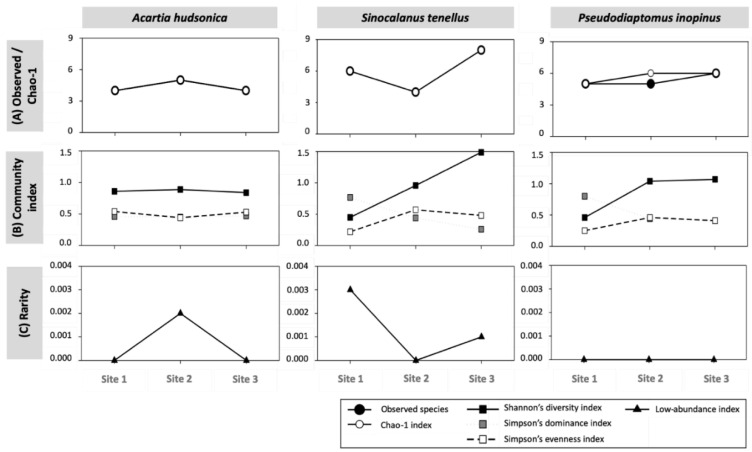
Comparison among bacterial alpha diversity indices of copepod species—*Acartia hudsonica*, *Sinocalanus tenellus*, and *Pseudodiaptomus inopinus*—by site. (**A**) Observed/Chao-1: observed species (black circle) and Chao-1 index (white circle), (**B**) community indices: Shannon’s diversity index (black rectangle), Simpson’s dominance and evenness indices (grey and white rectangles, respectively), (**C**) rarity: low-abundance index (black triangle).

The habitat environment changed, the composition of the copepod gut bacterial community and the community diversity changed, which showed different tendencies depending on the copepod species. The number of observed species in the copepod gut bacterial communities showed noticeable differences at Site 2, and tendencies differed for copepod species: increasing abundance from Sites 1 to 3 of *A. hudsonica* and *P. inopinus*, and a decreasing trend for *S. tenellus*. The Chao-1 index was mostly the same as the number of observed species, except for relatively higher richness for *P. inopinus* (Figure 4A), which is unlikely to indicate that any of the bacterial species present in the copepod gut will remain undetected.

The gut bacterial communities of *A. hudsonica* did not show notable differences in diversity, dominance, and evenness indices among study sites despite environmental changes such as salinity. As the suitability of the surrounding environment for S. *tenellus* decreased (based on salinity), its diversity index increased from Sites 1 to 3; however, the dominance index showed the opposite trend. The evenness index was highest for Site 2. The gut bacteria of *P. inopinus* showed the lowest diversity and evenness indices and highest dominance index at Site 1, which had the lowest salinity (Figure 4B). The differences in the gut bacterial community and diversity occurring within/between copepod species support that copepod species habitat environment and life history regulate gut bacterial community composition, playing a beneficial role in assisting the adsorption of bacteria for adaptation of continuously changing habitat environment [41,42,43].

Rarity based on the low-abundance index, which means the relative proportion of rare species based on the entire bacterial species detected, appeared in the gut flora of *A. hudsonica* at Site 2 and in *S. tenellus* at Sites 1 and 3 (relatively higher at Site 3). The species specifically detected in *A. hudsonica* at Site 2 and in *S. tenellus* at Sites 1 were Muribaculaceae. For *S. tenellus* at Sites 3, *Sporocytophaga* sp. and *Phaselicystis* sp. were specifically detected. There have been no studies on species that affect the rarity of gut bacterial communities in *A. hudsonica* and *S. tenellus*, with future studies looking at these species regarding copepod-associated bacteria required. For *P. inopinus*, the index value was calculated as zero, indicating that no rare species appeared in the gut bacterial community, regardless of the site (Figure 4C).

*Novosphingobium capsultum* was the dominant species in the gut bacteria of *A. hudsonica* at Sites 1 and 2. Subdominant bacteria were classified into the family Rhodobacteraceae. Conversely, the gut bacterial community at Site 3 was dominated by the family Rhodobacteraceae, with *N. capsultum* making a smaller contribution. These species belong to the class Alphaproteobacteria and accounted for most gut bacteria in *A. hudsonica* at approximately 95% or more regardless of study sites (Table 2A).

Different dominant/subdominant species were identified in *S. tenellus*. *Aeromonas hydrophila* belonging to the class Gammaproteobacteria of the phylum Proteobacteria was the dominant species at Site 1, and *Muribaculum* spp. belonging to the class Bacteroidia of the phylum Bacteroidetes was subdominant. Dominant/subdominant species at Site 2 were from the class Alphaproteobacteria of the phylum Proteobacteria (the family Rhodobacteraceae > *N. capsultum*). *Sporocytophaga* spp. in the class Cytophagia of the phylum Bacteroidetes were predominant at Site 3, and the subdominant species was *Bacillus velezensis* in the class Bacilli of the phylum Firmicutes. The dominant and subdominant species at Sites 1 and 2 accounted for most of the *S. tenellus* gut bacteria, over approximately 90%, but made up a relatively smaller proportion at Site 3 (61.5%) (Table 2B).

Except for the dominant species, *Polaribacter* sp. belonging to the class Flavobacteriia of the phylum Bacteroidetes made the largest contribution to gut bacteria from *P. inopinus* at Site 1, with other dominant or subdominant species belonging to class Alphaproteobacteria of the phylum Proteobacteria. The family Rhodobacteraceae and *N. capsulatum* were the subdominant bacterial species at Site 1 and were also identified as dominant and subdominant species at Site 3. *Brevundimonas denitrificans* was the dominant species at Site 3, followed by Rhodobacteraceae. These species accounted for more than 90% of the bacterial communities in *P. inopinus* at all sites (Table 2C).

In summary, there were some differences in the dominant/subdominant species in the copepod gut bacterial community depending on the copepod species and study sites; however, there were common trends, such as the abundance of Rhodobacteraceae in all three species (except Site 1, where the salinity level was lower than 10‰).

#### 3.2.2. Community Similarity and Core Bacterial Taxa

**Figure 5:**
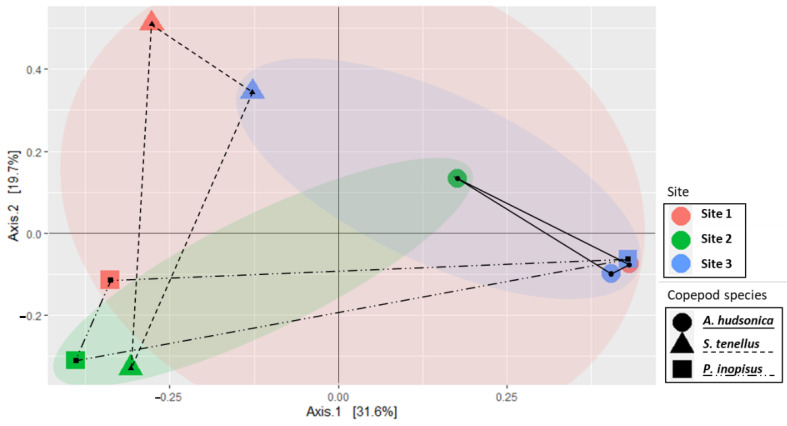
Biplot for a principal coordinate analysis (PCoA) of gut bacterial communities of *Acartia hudsonica* (circle, solid line), *Sinocalanus tenellus* (triangle, dotted line), and *Pseudodiaptomus inopinus* (rectangle, mixed line) at each site.

We confirmed the different tendencies of composition, dominant/subdominant species, and diversity indices of copepod gut bacterial communities depending on the different sites and copepod species. Similarity analysis was performed to estimate the similarity between inter- and intra-species-specific impacts on copepod gut bacterial communities. A clear distinction between copepod gut bacterial communities was observed in a PCoA biplot with the first coordinate accounting for 31.6% and the second for 19.7% of the variance. Bacterial communities were clustered according to the sampling sites and the copepod species. Therefore, similarity showed differences caused by inter- and intra-species-specific impacts (Figure 5).

Based on Axis 1, the similarity of bacterial community has been shown to be high between *S. tenellus* and *P. inopinus* compared to *A. hudsonica*. These differences in gut bacterial community composition among the target copepod species are considered as the results due to phylogenetic relationship. The gut bacterial communities of aquatic organisms, including invertebrates such as Bivalvia, as well as vertebrates, are strongly related to phylogeography and can be changed or maintained during the evolutionary process [12,44]. In the case of target copepod species in this study, Acartiidae (*A. hudsonica*) received relatively low nodal support on trees with Centropagidae (*S. tenellus*) and Pseudodiaptomidae (*P. inopinus*) using morphological data and parsimony-based phylogeny [45].

Meanwhile, even the same/phylogenetically similar copepod species differed in the similarity of the gut bacterial community composition depending on their habitat environment. The bacterial community of *A. hudsonica* displayed the highest similarity, except for Site 2 where *Muribaculum* sp. was specifically detected. In contrast, *S. tenellus* had relatively low similarity because *Bacillus velezensis*, *Aeromonas hydrophila*, and *Muribaculum* sp. identified at other sites were not detected at Site 2. *P. inopinus* showed high variability because Rhodobacteraceae and *Bradyrhizobium cytisi* were detected in greater quantities at Site 3 compared with at the other sites. *A. hudsonica* is omnivorous and mainly feeds on phytoplankton, rotifers, and ciliates [46]. *S. tenellus* is also omnivorous and a suspension feeder, preferring small-sized plankton [47]. In contrast, *P. inopinus* is a detritivore which prefers small particles [48]. Based on these feeding characteristics, target copepod species can influence the composition of gut bacterial community through their feeding activities. However, the composition of food environment may vary depending on the water quality factors such as salinity at each habitat [24], so if the food sources are changed, even if it is the same copepod species, the gut bacterial community can be also changed.

On the contrary, some results showed that different copepod species had relatively similar patterns in the same habitat environment (*S. tenellus*–*P. inopinus* at Site 1; *A. hudsonica*–*P. inopinus* at Site 3). These tendencies may be the results that the gut bacterial community of copepod species was also affected by the free-living bacterial community in surrounding water, which is similar to the results of a previous study on zooplankton-associated bacterial communities in estuarine ecosystems [44].

#### 3.2.3. Core Bacterial Species in Copepod Gut and Free-Living Bacterial Communities

**Figure 6:**
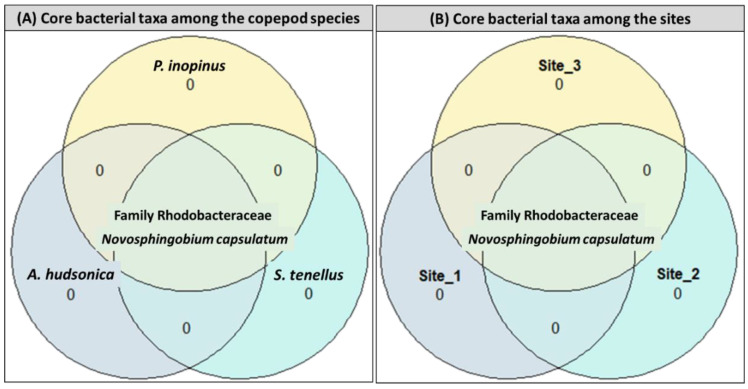
Shared and unique core taxa in the gut bacterial communities among (**A**) copepods (*Acartia hudsonica*, *Sinocalanus tenellus*, and *Pseudodiaptomus inopinus*) and (**B**) sites using Venn diagrams; core taxa were selected based on 0.001 detection probability in at least 90% samples and prevalence = 0.75.

Analysis of the bacterial taxa in the gut of copepods uniquely identified at each site/copepod or at more than two sites/copepod species’ two core bacteria, *Novosphingobium capsulatum* and the family Rhodobacteraceae, which were shared among the targeted copepod species *A. hudsonica*, *S. tenellus*, and *P. inopinus* and all sites (Figure 6). These species are classified as Alphaproteobacteria of the phylum Proteobacteria and were identified as dominant or subdominant species with a high proportion of the entire bacterial community at almost all sites (Table 2, Table S2 and Table S3).

The interaction of the bacterial class Alphaproteobacteria, which is abundant in seawater and freshwater aquatic ecosystems as a zooplankton-associated bacterial community, with copepods has been known for its relatively high resolution [41,49]. The family Rhodobacteraceae, which is one of the most common marine bacterial groups and is highly abundant in marine pelagic and sediments, is the species that forms algae-associated biofilm [50,51]. Based on the characteristics of Rhodobacteraceae, they were identified as a core species of the gut bacterial community in relation to phytoplankton feeding of copepods. We could not identify these further to the genus or species level. The bacterial genus *Novosphingobium* is often associated with the biodegradation of aromatic compounds and is distributed over estuarine and coastal sediments, and marine aquatic environments exposed to high levels of anthropogenic impacts [52]. As previous findings on *Novosphingobium* sp. were mainly the perspective of the aquatic free-living bacterial community, further research is required to understand the role of *N. capsulatum* as a core species in the copepod gut bacterial community.

## 4. Conclusions

We detected the gut bacterial communities of brackish copepods, *A. hudsonica*, *S. tenellus*, and *P. inopinus*, among different sites in the Saemangeum reservoir using NGS technology. To extract bacterial DNA from small-sized copepod species, we designed a pretreatment method using whole copepod individuals without dissection. The core species were *N. capsulatum* and the family Rhodobacteraceae belonging to Alphaproteobacteria. Their OTU proportions were 24.2% and 22.5% in the bacterial species detected from all copepod species at all points, respectively. The core bacterial species have a common property of decomposing organic substrates; therefore, they are important species for metabolic activities such as digestion. We found inter- and intra-species-specific differences in the various compositions among species and sites. These results support that environmental variables and feeding behaviour of the target brackish copepods affect their gut bacterial communities. In the present study, we briefly examined the brackish bacterial compositions of the dominant brackish copepods. Through our approach, further studies regarding the interactions among gut-dwelling bacteria and consumed food items (e.g., phytoplankton) and environmental factors are important topics to be considered for a better understanding of copepod-associated microbial food web dynamics.

## Figures and Tables

**Table 1:** Summary of environmental characteristics—water depths, water quality variables and phytoplankton community composition—at each study site.

Factors			Site 1	Site 2	Site 3
Water quality	Water depths	(m)	2	4.5	22.5
	Temperature	°C	14.6	13.7	13.0
	Salinity	(‰)	4.1	5.1	12.9
	pH		8.4	8.5	8.4
	Dissolved Oxygen	(mg/L)	10.7	11.1	9.2
	Chemical Oxygen Demand	(mg/L)	5.2	8.4	6.8
	Electrical Conductivity	(S/m)	5898	6795	15,665
	Chlorophyll-*a*	(μg/L)	5.4	7.0	4.9
	Total Nitrogen	(mg/L)	3.391	3.023	1.240
	Total Phosphorus	(mg/L)	0.084	0.039	0.045
Phytoplankton composition	Chlorophyta	(%)	38.75	24.82	7.32
	Bacillariophyta	(%)	61.25	75.08	92.68

**Table 2:** Dominant and subdominant species of copepod gut bacterial communities based on the proportion of the operational taxonomic units (OTUs)—(**A**) *Acartia hudsonica*, (**B**) *Sinocalanus tenellus*, and (**C**) *Pseudodiaptomus inopinus.*

Sample		Dominant and Subdominant Bacterial Species			
		Phylum	Class	Family/Genus/Species	OTUs (%)
(A) *A. hudsonica*	Site 1	Proteobacteria	Alphaproteobacteria	*Novosphingobium capsulatum*	752 (48.9)
				Rhodobacteraceae	725 (47.1)
	Site 2	Proteobacteria	Alphaproteobacteria	*N. capsulatum*	6813 (51.1)
				Rhodobacteraceae	5799 (43.5)
	Site 3	Proteobacteria	Alphaproteobacteria	Rhodobacteraceae	671 (54.1)
				*N. capsulatum*	524 (42.2)
(B) *S. tenellus*	Site 1	Proteobacteria	Gammaproteobacteria	*Aeromonas hydrophila*	13,024 (86.7)
		Bacteroidetes	Bacteroidia	*Muribaculum* sp.	1366 (9.1)
	Site 2	Proteobacteria	Alphaproteobacteria	Rhodobacteraceae	54 (54.5)
				*N. capsulatum*	36 (36.4)
	Site 3	Bacteroidetes Firmicutes	Cytophagia Bacilli	*Sporocytophaga* sp.	1546 (37.3)
				*Bacillus velezensis*	1005 (24.2)
(C) *P. inopinus*	Site 1	Bacteroidetes	Flavobacteriia	*Polaribacter* sp.	41 (61.2)
		Proteobacteria	Alphaproteobacteria	Rhodobacteraceae	12 (17.9)
				*N. capsulatum*	
	Site 2	Proteobacteria	Alphaproteobacteria	*Brevundimonas denitrificans*	373 (89.2)
				Rhodobacteraceae	25 (6.0)
	Site 3	Proteobacteria	Alphaproteobacteria	Rhodobacteraceae	1131 (48.6)
				*N. capsulatum*	958 (41.2)

## Data Availability

DNA sequences deposited in https://doi.org/10.5061/dryad.66t1g1k1k.

## Supplementary Materials

The following are available online at https://www.mdpi.com/2076-2615/11/2/542/s1, Table S1: Summary of copepods gut bacterial communities identified from *Acartia hudsonica*, *Sinocalanus tenellus*, and *Pseudodiaptomus inopinus* at each site, Table S2: Summary of (A) common species and (B) unique species of the gut bacterial communities among/in the copepod species (*Acartia hudsonica*, *Sinocalanus tenellus*, and *Pseudodiaptomus inopinus*). Composition (%) was calculated based on all species comprising the gut bacterial community identified from the targeted copepods, Table S3: Summary of (A) common species and (B) unique species of the copepod gut bacterial communities among/in Sites 1, 2, and 3. Composition (%) was calculated based on all species comprising the copepod gut bacterial community identified from the studied sites.

## References

[1] Rakhesh M., Raman A.V., Ganesh T., Chandramohan P., Dehairs F. (2013). Small copepods structuring mesozooplankton community dynamics in a tropical estuary-coastal system. Estuar. Coast. Shelf Sci..

[2] Thor P., Dam H.G., Rogers D.R. (2003). Fate of organic carbon released from decomposing copepod fecal pellets in relation to bacterial production and ectoenzymatic activity. Aquat. Microb. Ecol..

[3] Møller E.F., Thor P., Nielsen T.G. (2003). Production of DOC by *Calanus finmarchicus*, *C. glacialis* and *C. hyperboreus* through sloppy feeding and leakage from fecal pellets. Mar. Ecol. Prog. Ser..

[4] Chevalier C., Stojanović O., Colin D.J., Suarez-Zamorano N., Tarallo V., Veyrat-Durebex C., Rigo D., Fabbiano S., Stevanović A., Hagemann S. (2015). Gut microbiota orchestrates energy homeostasis during cold. Cell.

[5] Macke E., Callens M., De Meester L., Decaestecker E. (2017). Host-genotype dependent gut microbiota drives zooplankton tolerance to toxic cyanobacteria. Nat. Commun..

[6] Tang K.W., Turk V., Grossart H.-P. (2010). Linkage between crustacean zooplankton and aquatic bacteria. Aquat. Microb. Ecol..

[7] Glud R.N., Grossart H.P., Larsen M., Tang K.W., Arendt K.E., Rysgaard S. (2015). Copepod carcasses as microbial hot spots for pelagic denitrification. Limnol. Oceanogr..

[8] Stief P., Lundgaard A.B., Morales-Ramirez A., Thamdrup B., Glud R.N. (2017). Fixed-nitrogen loss associated with sinking zooplankton carcasses in a coastal oxygen minimum zone (Golfo Dulce, Costa Rica). Front. Mar. Sci..

[9] Nuester J., Shema S., Vermont A., Fields D.M., Twining B.S. (2014). The regeneration of highly bioavailable iron by meso- and microzooplankton. Limnol. Oceanogr..

[10] Schmidt K., Schlosser C., Atkinson A., Fielding S., Venables H.J., Waluda C.M., Achterberg E.P. (2016). Zooplankton gut passage mobilizes lithogenic iron for ocean productivity. Curr. Biol..

[11] Li Y., Xu Z., Liu H. (2021). Nutrient-imbalanced Conditions Shift the Interplay Between Zooplankton and Gut Microbiota. BMC Bioinform..

[12] Mioduchowska M., Zając K., Bartoszek K., Madanecki P., Kur J., Zając T. (2020). 16S rRNA-based metagenomic analysis of the gut microbial community associated with the DUI species *Unio crassus* (Bivalvia: Unionidae). J. Zoolog. Syst. Evol. Res..

[13] Su S., Munganga B.P., Du F., Yu J., Li J., Yu F., Wang M., He X., Li X., Bouzoualegh R. (2020). Relationship between the fatty acid profiles and gut bacterial communities of the Chinese Mitten Crab (*Eriocheir sinensis*) from ecologically different habitats. Front. Microbiol..

[14] Jing T.Z., Qi F.H., Wang Z.Y. (2020). Most dominant roles of insect gut bacteria: Digestion, detoxification, or essential nutrient provision?. Microbiome.

[15] Muratore M., Sun Y., Prather C. (2020). Environmental nutrients alter bacterial and fungai gut microbiomes in the common medow katydid, *Orchelimum vulgare*. Front. Microbiol..

[16] Datta M.S., Almada A.A., Baumgartner M.F., Mincer T.J., Tarrant A.M., Polz M.F. (2018). Inter-individual variability in copepod microbiomes reveals bacterial networks linked to host physiology. ISME J..

[17] Grossart H.-P., Dziallas C., Leunert F., Tang K.W. (2010). Bacteria dispersal by hitchhiking on zooplankton. Proc. Natl. Acad. Sci. USA.

[18] Shoemaker K.M., Duhamel S., Moisander P.H. (2019). Copepods promote bacterial community changes in surrounding seawater through farming and nutrient enrichment. Environ. Microbiol..

[19] Ho T.W., Hwang J.S., Cheung M.K., Kwan H.S., Wong C.K. (2017). DNA-based study of the diet of the marine calanoid copepod *Calanus sinicus*. J. Exp. Mar. Biol. Ecol..

[20] Hirai J., Hamamoto Y., Honda D., Hidaka K. (2018). Possible aplanochytrid (Labyrinthulea) prey detected using 18S metagenetic diet analysis in the key copepod species *Calanus sinicus* in the coastal waters of the subtropical western North Pacific. Plankton Benthos Res..

[21] Yeh H.D., Questel J.M., Maas K.R., Bucklin A. (2020). Metabarcoding analysis of regional variation in gut contents of the copepod *Calanus finmarchicus* in the North Atlantic Ocean. Deep. Res. Part II Top. Stud. Oceanogr..

[22] Oh H.J., Krogh P.H., Jeong H.G., Joo G.J., Kwak I.S., Hwang S.J., Gim J.S., Chang K.H., Jo H. (2020). Pretreatment method for DNA barcoding to analyze gut contents of rotifers. Appl. Sci..

[23] Majumder S., Dhua R.P., Kar S., Mishra T., Mahapatra S.S., Shit S., Patra A. (2015). Zooplankton diversity influenced by hydro biological parameters in some ponds of south eastern part of Bankura town of WB, India. Int. J. Adv. Res..

[24] Oda Y., Nakano S., Suh J.M., Oh H.J., Jin M.Y., Kim Y.J., Chang K.H. (2018). Spatiotemporal variability in a copepod community associated with fluctuations in salinity and trophic state in an artificial brackish reservoir at Saemangeum, Korea. PLoS ONE.

[25] Lie H.J., Cho C.H., Lee S., Kim E.S., Koo B.J., Noh J.H. (2008). Changes in marine environment by a large coastal development of the Saemangeum reclamation project in Korea. Ocean. Polar Res..

[26] Ryu J., Khim J.S., Choi J.W., Shin H.C., An S., Park J. (2011). Environmentally associated spatial changes of a macrozoobenthic community in the Saemangeum tidal flat, Korea. J. Sea Res..

[27] Lee H.J., Lee S.H. (2012). Geological consequences of the Saemangeum Dyke, mid–west coast of korea: A review. Ocean. Sci. J..

[28] Ministry of Environment (2018). Water Pollution Process Standard.

[29] Ministry of Oceans and Fisheries (2018). Marine Environmental Process Standards.

[30] Hendey N.I. (1974). The permanganate method for cleaning using diatoms. Nova Hedwig. Beih..

[31] Bennke C.M., Pollehne F., Müller A., Hansen R., Kreikemeyer B., Labrenz M. (2018). The distribution of phytoplankton in the Baltic Sea assessed by a prokaryotic 16S rRNA gene primer system. J. Plankton Res..

[32] Edgar R.C. (2010). Search and clustering orders of magnitude faster than BLAST. Bioinformatics.

[33] Altschul S.F., Gish W., Miller W., Myers E.W., Lipman D.J. (1990). Basic local alignment search tool. J. Mol. Biol..

[34] Caporaso J.G., Kuczynski J., Stombaugh J., Bittinger K., Bushman F.D., Costello E.K., Fierer N., Peña A.G., Goodrich J.K., Gordon J.I. (2010). QIIME allows analysis of high-throughput community sequencing data. Nat. Methods.

[35] Lahti L., Shetty S. Tools for Microbiome Analysis in R. Version 1.5.28. 2017. http://microbiome.github.com/microbiome.

[36] Lance J. (1965). Respiration and osmotic behaviour of the copepod *Acartia tonsa* in diluted sea water. Comp. Biochem. Physiol..

[37] Tester P.A., Turner J.T. Why is *Acartia tonsa* restricted to estuarine habitats. Proceedings of the Fourth International Conference Copepoda.

[38] Cervetto G., Gaudy R., Pagano M. (1999). Influence of salinity on the distribution of *Acartia tonsa* (Copepoda, Calanoida). J. Exp. Mar. Biol. Ecol..

[39] Lu K., Lu Y., Lin X., Zheng Z., Yao G. (2001). Effects of the experimental factors on filtering and grazing rates of *Sinocalanus tenellus*. Mar. Sci. Haiyang Kexue.

[40] Cordell J.R., Rassmussen M., Bollens S.M. (2007). Biology of the invasive copepod *Pseudodiaptomus inopinus* in a northeast Pacific estuary. Mar. Ecol. Prog. Ser..

[41] Grossart H.P., Dziallas C., Tang K.W. (2009). Bacterial diversity associated with freshwater zooplankton. Environ. Microbiol. Rep..

[42] Bickel S.L., Tang K.W., Grossart H.P. (2014). Structure and function of zooplankton-associated bacterial communities in a temperate estuary change more with time than with zooplankton species. Aquat. Microb. Ecol..

[43] Tang K., Dziallas C., Hutalle-Schmelzer K., Grossart H.P. (2009). Effects of food on bacterial community composition associated with the copepod *Acartia tonsa* Dana. Biol. Lett..

[44] Bickel S.L., Tang K.W. (2014). Carbon substrate usage by zooplankton-associated bacteria, phytoplankton-associated bacteria, and free-living bacteria under aerobic and anaerobic conditions. Mar. Biol..

[45] Bradford-Grieve J.M., Boxshall G.A., Ahyong S.T., Ohtsuka S. (2010). Cladistic analysis of the calanoid copepoda. Invertebr. Syst..

[46] Rollwagen Bollens G.C., Penry D.L. (2003). Feeding dynamics of *Acartia* spp. copepods in a large, temperate estuary (San Francisco Bay, CA). Mar. Ecol. Prog. Ser..

[47] Hada A., Uye S. (1991). Cannibalistic feeding behavior of the brackish-water copepod *Sinocalanus tenellus*. J. Plankton Res..

[48] Chen M., Kim D., Liu H., Kang C.K. (2018). Variability in copepod trophic levels and feeding selectivity based on stable isotope analysis in Gwangyang Bay of the southern coast of the Korean Peninsula. Biogeosciences.

[49] De Corte D., Lekunberri I., Sintes E., Garcia J.A.L., Gonzales S., Herndl G.J. (2014). Linkage between copepods and bacteria in the North Atlantic Ocean. Aquat. Microb. Ecol..

[50] Haggerty J.M., Dinsdale E.A. (2017). Distinct biogeographical patterns of marine bacterial taxonomy and functional genes. Glob. Ecol. Biogeogr..

[51] Pohlner M., Dlugosch L., Wemheuer B., Mills H., Engelen B., Reese B.K. (2019). The majority of active Rhodobacteraceae in marine sediments belong to uncultured genera: A molecular approach to link their distribution to environmental conditions. Front. Microbiol..

[52] Gan H.M., Hudson A.O., Rahman A.Y.A., Chan K.G., Savka M.A. (2013). Comparative genomic analysis of six bacteria belonging to the genus Novosphingobium: Insights into marine adaptation, cell-cell signaling and bioremediation. BMC Genom..

