# Supplementary material for: Application of Next-Generation Sequencing for the Determination of the Bacterial Community in the Gut Contents of Brackish Copepod Species (Acartia hudsonica, Sinocalanus tenellus, and Pseudodiaptomus inopinus)
Source: Animals (Basel). 2021 Feb 19;11(2):542. doi: 10.3390/ani11020542 (PMC7923213; doi:10.3390/ani11020542)
Supplement: Supplementary file 1 [file animals-11-00542-s001.pdf]

animals

Supplementary Materials: Table S1: Summary of copepods gut bacterial communities identified from *Acartia hudsonica*, *Sinocalanus tenellus*, and *Pseudodiaptomus inopinus* in each site., Table S2: Summary of **(A)** common species and **(B)** unique species of the gut-bacterial communities among/in the copepod species (*Acartia hudsonica*, *Sinocalanus tenellus*, and *Pseudodiaptomus inopinus*). Composition (%) was calculated based on the all species that make up the gut-bacterial community identified from the copepods we targeted., Table S3: Summary of **(A)** common species and **(B)** unique species of the copepods gut-bacterial communities among/in the sites 1, 2, and 3. Composition (%) was calculated based on the all species that make up the copepods gut bacterial community identified from the sites we studied.

Supplementary Materials Legends

Table S1. Summary of copepods gut bacterial communities identified from *Acartia hudsonica*, *Sinocalanus tenellus*, and *Pseudodiaptomus inopinus* in each site.
Table S2. Summary of **(A)** common species and **(B)** unique species of the gut-bacterial communities among/in the copepod species (*Acartia hudsonica*, *Sinocalanus tenellus*, and *Pseudodiaptomus inopinus*). Composition (%) was calculated based on the all species that make up the gut-bacterial community identified from the copepods we targeted.
Table S3. Summary of **(A)** common species and **(B)** unique species of the copepods gut-bacterial communities among/in Sites 1, 2 and 3. Composition (%) was calculated based on the all species that make up the copepods gut-bacterial community identified from the sites we studied.

Table S1. Summary of copepods gut bacterial communities identified from *Acartia hudsonica*, *Sinocalanus tenellus*, and *Pseudodiaptomus inopinus* in each site.

Sample		Bacterial community		Accession number	Identities (%)	Number of reads
		Phylum	Species/Genus/Family			
A. hudsonica	Site 1	Firmicutes	*Bacillus velezensis*	NR_075005.2	99	25
		Proteobacteria	*Novosphingobium capsulatum*	NR_113591.1	98	752
			Rhodobacteraceae	NR_159237.1	89	725
			Bradyrhizobium cytisi	NR_116360.2	99	36
	Site 2	Bacteroidetes	Muribaculaceae	NR_144616.1	89	22
			Sediminibacterium roseum	NR_159130.1	97	93
		Proteobacteria	*Novosphingobium capsulatum*	NR_113591.1	98	6813
			Rhodobacteraceae	NR_159237.1	89	5799
			Bradyrhizobium cytisi	NR_116360.2	99	596
	Site 3	Bacteroidetes	*Sediminibacterium roseum*	NR_159130.1	97	16
		Proteobacteria	*Novosphingobium capsulatum*	NR_113591.1	98	524
			Rhodobacteraceae	NR_159237.1	89	671
			Bradyrhizobium cytisi	NR_116360.2	99	30
S. tenellus	Site 1	Bacteroidetes	*Muribaculum* sp.	NR_144616.1	90	1792
		Firmicutes	*Bacillus velezensis*	NR_075005.2	99	70
		Proteobacteria	*Aeromonas hydrophila*	NR_074841.1	99	13024
			Novosphingobium capsulatum	NR_113591.1	98	24
			Rhodobacteraceae	NR_159237.1	89	27
			Brevundimonas bullata	NR_113611.1	99	79
	Site 2	Proteobacteria	*Novosphingobium capsulatum*	NR_113591.1	98	36
			Rhodobacteraceae	NR_159237.1	89	54
			Bradyrhizobium cytisi	NR_116360.2	99	2
		Planctomycetes	Pirellulaceae	NR_043384.1	85	7
	Site 3	Bacteroidetes	*Sporocytophaga* sp.	NR_025463.1	93	1546
			Muribaculum sp.	NR_144616.1	91	10

		Firmicutes	*Bacillus velezensis*	NR_075005.2	99	1005
		Proteobacteria	*Aeromonas hydrophila*	NR_074841.1	99	369
			Novosphingobium capsulatum	NR_113591.1	98	94
			Rhodobacteraceae	NR_159237.1	89	153
			Phaselicystis sp.	NR_044523.1	90	970
			Bradyrhizobium cytisi	NR_116360.2	99	3
P. inopinus	Site 1	Proteobacteria	*Novosphingobium capsulatum*	NR_113591.1	98	11
			Rhodobacteraceae	NR_159237.1	89	25
			Bradyrhizobium cytisi	NR_116360.2	99	1
			Brevundimonas denitrificans	NR_133989.1	99	373
			Brevundimonas bullata	NR_113611.1	99	8
	Site 2	Bacteroidetes	*Polaribacter* sp.	NR_153703.1	95	41
			Sediminibacterium roseum	NR_159130.1	97	1
		Proteobacteria	*Novosphingobium capsulatum*	NR_113591.1	98	12
			Rhodobacteraceae	NR_159237.1	89	12
		Planctomycetes	Pirellulaceae	NR_043384.1	85	1
	Site 3	Bacteroidetes	*Polaribacter* sp.	NR_153703.1	95	82
		Proteobacteria	*Novosphingobium capsulatum*	NR_113591.1	98	958
			Rhodobacteraceae	NR_159237.1	89	1131
			Bradyrhizobium cytisi	NR_116360.2	99	110
			Hydrogenophaga taeniospiralis	NR_114131.1	99	13
		Planctomycetes	Pirellulaceae	NR_043384.1	85	32

Table S2. Summary of **(A)** common species and **(B)** unique species of the gut-bacterial communities among/in the copepod species (*Acartia hudsonica*, *Sinocalanus tenellus*, and *Pseudodiaptomus inopinus*). Composition (%) was calculated based on the all species that make up the gut-bacterial community identified from the copepods we targeted.

Sample		Bacterial species			
		Phylum	Class	Family/Genus/Species	Composition (%)
**(A)** Common species		Bacteroidetes	Bacteroidia	Muribaculaceae	3.56
			Chitinophagia	*Sediminibacterium roseum*	0.43
			Flavobacteriia	*Sporocytophaga* sp.	2.98
		Firmicutes	Bacilli	*Bacillus velezensis*	2.14
		Planctomycetes	Planctomycetia	Pirellulaceae	0.03
		Proteobacteria	Alphaproteobacteria	*Brevundimonas bullta*	0.16
				Bradyrhizobium cytisi	2.61
				Rhodobacteraceae	28.49
				Novosphingobium capsulatum	31.53
			Deltaproteobacteria	*Phaselicystis* sp.	1.87
			Gammaproteobacteria	*Aeromonas hydrophila*	35.08
**(B)** Unique species	*A. hudsonica*	There is no unique bacterial species.			
	S. tenellus	There is no unique bacterial species.			
	P. inopinus	Bacteroidetes	Flavobacteriia	*Polaribacter* sp.	0.08
		Proteobacteria	Alphaproteobacteria	*Brevundimonas denitrificans*	0.24
			Betaproteobacteria	*Hydrogenophaga taeniospiralis*	0.01

Table S3. Summary of **(A)** common species and **(B)** unique species of the copepods gut-bacterial communities among/in Sites 1, 2 and 3. Composition (%) was calculated based on the all species that make up the copepods gut-bacterial community identified from the sites we studied.

Sample		Bacterial species			
		Phylum	Class	Family/Genus/Species	Composition (%)
**(A)** Common species		Bacteroidetes	Bacteroidia	Muribaculaceae	4.78
			Chitinophagia	*Sediminibacterium roseum*	0.29
			Flavobacteriia	*Polaribacter* sp.	0.32
		Firmicutes	Bacilli	*Bacillus velezensis*	2.88
		Planctomycetes	Planctomycetia	Pirellulaceae	0.10
		Proteobacteria	Alphaproteobacteria	*Bradyrhizobium cytisi*	2.04
				Rhodobacteraceae	22.52
				Novosphingobium capsulatum	24.16
			Gammaproteobacteria	*Aeromonas hydrophila*	35.08
**(B)** Unique species	Site 1	Proteobacteria	Alphaproteobacteria	*Brevundimonas bullata*	0.23
				Brevundimonas denitrificans	0.98
	Site 2	There is no unique bacterial species.			
	Site 3	Bacteroidetes	Cytophagia	*Sporocytophaga* sp.	4.05
		Proteobacteria	Deltaproteobacteria	*Phaselicystis* sp.	2.54
			Betaproteobacteria	*Hydrogenophaga taeniospiralis*	0.03
